# Comparing deep belief networks with support vector machines for classifying gene expression data from complex disorders

**DOI:** 10.1002/2211-5463.12652

**Published:** 2019-06-07

**Authors:** Johannes Smolander, Matthias Dehmer, Frank Emmert‐Streib

**Affiliations:** ^1^ Predictive Society and Data Analytics Lab Faculty of Information Technology and Communication Sciences Tampere University Finland; ^2^ Turku Centre for Biotechnology University of Turku Finland; ^3^ Institute for Intelligent Production Faculty for Management University of Applied Sciences Upper Austria Steyr Austria; ^4^ Department of Mechatronics and Biomedical Computer Science UMIT Hall in Tyrol Austria; ^5^ College of Computer and Control Engineering Nankai University Tianjin China; ^6^ Institute of Biosciences and Medical Technology Tampere Finland

**Keywords:** artificial intelligence, deep belief network, deep learning, genomics, neural networks, support vector machine

## Abstract

Genomics data provide great opportunities for translational research and the clinical practice, for example, for predicting disease stages. However, the classification of such data is a challenging task due to their high dimensionality, noise, and heterogeneity. In recent years, deep learning classifiers generated much interest, but due to their complexity, so far, little is known about the utility of this method for genomics. In this paper, we address this problem by studying a computational diagnostics task by classification of breast cancer and inflammatory bowel disease patients based on high‐dimensional gene expression data. We provide a comprehensive analysis of the classification performance of deep belief networks (DBNs) in dependence on its multiple model parameters and in comparison with support vector machines (SVMs). Furthermore, we investigate combined classifiers that integrate DBNs with SVMs. Such a classifier utilizes a DBN as representation learner forming the input for a SVM. Overall, our results provide guidelines for the complex usage of DBN for classifying gene expression data from complex diseases.

**Abbreviations**
ANN: artificial neural network
Bprop: backpropagation
CD: contrastive divergence
CNN: convolutional neural networks
DBN: deep belief networks
DL: deep learning
IBD: inflammatory bowel disease
MLPs: multilayer perceptrons
RBM: restricted Boltzmann machine
RNNs: recurrent neural networks
Rprop: resilient backpropagation
SE1DCNN: sample expansion‐based 1DCNN
SGD: stochastic gradient descent
SVM: support vector machines

Technological progress in the generation of genome‐scale high‐throughput data has led to a flood of data on the DNA, RNA, and protein levels 1. These data provide new and exciting opportunities for studying molecular mechanisms to enhance our understanding in basic biology and medicine 2, 3, 4, 5. Particularly for the latter field, new avenues open toward a personalized or precision medicine, both heavily based on genomic medicine 6, 7, 8, 9. However, challenges for the analysis of such data, for example, for classifying disease stages of patients, are their high dimensionality, noise, and the heterogeneity of the underlying patient samples, especially for gene expression data. For this reason, the major purpose of this paper was to investigate deep learning (DL) classifiers for the computational diagnostics of two complex disorders, breast cancer and inflammatory bowel disease (IBD), based on gene expression data.

Deep learning is a new methodology currently receiving much attention 10. DL corresponds to a set of learning algorithms that can be used to learn complex representations, for example, via multilayer neural networks with many hidden units 11, 12. So far, DL has been successfully applied to many problems where it achieved excellent results. For instance, a DL method set a new record for the classification of handwritten digits of the MNIST data set with an error rate of 0.21% 13. Further application areas are image recognition 10, 11, 14, speech recognition 15, natural language understanding 16, and acoustic modeling 17. Also in computational biology, DL has been used for analyzing DNA data 18, 19, 20. For instance, in molecular biology regulatory mechanisms have been studied, for example, for understanding forms of alternative splicing or predicting protein binding sites. However, very little is known about analyzing gene expression data 21. Only recently 22 investigated the tumor classification of different cancers by introducing methods called sample expansion‐Based SAE and sample expansion‐based 1DCNN (SE1DCNN), both based on autoencoders. Unfortunately, their analysis was conducted for very small data sets making the statistical interpretation difficult. It is revealing that a recent review by 23 does not provide one example for the classification of gene expression data by any DL method and the review by 24 merely mentioned the study by 21. This illustrates the current lack of understanding about DL in genomics.

In this paper, we will study deep belief network (DBN), a particular form of DL methods. A DBN is an artificial neural network (ANN) model that is trained in two phases. In the first phase, called pretraining, a restricted Boltzmann machine (RBM) is used to initialize the network model. This phase is unsupervised. In the second phase, called fine‐tuning, this model is then processed in a supervised manner. We examine two algorithms for computing the error gradients of stochastic gradient descent (SGD), used for optimizing the model in the fine‐tuning phase. These two algorithms are called backpropagation (Bprop) and resilient backpropagation (Rprop), whereas the latter is a more efficient advancement of Bprop 25. In addition, we examine autoencoders that are learned by a similar two‐phase process 26. For reasons of comparison, we study support vector machines (SVMs) using the efficient LIBSVM implementation 27.

Deep learning methods are known to be very complex models compared to conventional methods, for example, SVMs or random forests 28. This complexity comes with respect to the choice of the available model parameters (architecture of the neural network, number of neurons per unit, learning rates, etc.) but also the required computational resources for their execution, usually, demanding the usage of a computer cluster – as is needed for our analysis. In order to obtain insights into the working mechanisms of DL methods for the diagnostic classification of gene expression data, we perform comprehensive analyses centered around DBNs. Major aspects of our investigations include studying the influence of the network architecture, choice of the algorithm for the fine‐tuning phase, and regularization methods.

A second major objective of this paper was to investigate the integration of DBNs with SVMs. Put simply, this means we are using a hidden unit of the learned network structure as input layer for a SVM. This can be seen as a feature selection mechanism for the SVM because the DBN is used as a representation learner. Specifically, we investigate the integration of DBN with Bprop and SVM, DBN with Rprop and SVM, RBM‐learned representations and SVM, and autoencoder‐learned representations and SVM.

In order to obtain robust results, for our analysis we are using two gene expression data sets for complex disorders: (a) breast cancer and (b) IBD. In contrast to single‐gene disorders, for example, sickle cell anemia or cystic fibrosis, complex or multifactorial disorders are caused by the synergy of genetic, environmental, and lifestyle factors 29, 30. One common property of complex disorders is that the genetic predisposition is inheritable, but the development is determined by the lifestyle and environment of individuals. Another feature is that the predisposition or susceptibility is determined by multiple genes, sometimes by hundreds. Cancers are different from most complex disorders in that most of them are nonhereditary (sporadic) cancers. In contrast, hereditary cancers are caused by mutations in DNA repair genes, whereas most of the sporadic cancers have currently an indefinite molecular basis for their genetic instability that promotes their development 31. Also IBD is a complex disorder. Two of its main subtypes are ulcerative colitis (UC) and Crohn's disease. In our analysis, we will study the classification of both IBD types, also in combination with samples from control patients.

In contrast to previous investigations analyzing DL for genomics data, our study is different with respect to the following points. First, we are using gene expression data from DNA microarray experiments, which are currently understudied in genomics. This complements studies using DNA sequence data, for example, 18, 19, 20. Second, the sample size of the data sets we are studying is sufficiently large allowing to obtain statistically robust results. In genomics, this does not hold for every data set, especially, in a clinical context when the data are derived from patient – as is the case for our data sets. Third, we study the integration of a DBN with a SVM. This complements studies focusing on either of these classifiers in isolation or using nongenomic data 16. Our results will provide guidelines for the complex usage of DL methods for diagnosing gene expression data from breast cancer and IBD patients.

Our paper is organized as follows. In the next section, we present the methods we use to analyze the data. Then, we present our results and a discussion thereof. We finish this paper with concluding remarks.

## Methods

### Deep learning models

There are a number of different learning algorithms available that can be used to build DL models for supervised learning problems. Examples for such models are convolutional neural networks (CNNs), DBNs, multilayer perceptrons (MLPs) and recurrent neural networks (RNNs) 11, 32. Each of these four models could be used to build a supervised DL model. In the following, we discuss them briefly and explain why we selected a DBN for our analysis.

Currently, CNNs are the dominating model for tasks involving computer vision 11. CNNs are particularly effective in situations where the data consist of multiple arrays and nearby values of data arrays are correlated with each other, as can be found in images, videos, or sound data. Originally, CNNs were developed to simulate the visual cortex of humans and CNNs take advantage of the properties exhibited by natural signals. The name ‘convolution’ indicates that CNNs apply mathematical convolution operations for the processing of information.

Recurrent neural networks are commonly used in tasks involving sequential input data, such as speech data, music, or text data 33. Also, such a sequential input implies a certain correlation structure between the input data because the order of the data is fixed and cannot be arbitrarily chosen. In contrast to MLPs and CNNs which are feedforward networks, RNNs are recurrent networks containing cycles and feedback loops. This makes them potentially more complex models than feedforward neural networks, but introduces also problems making them more difficult to handle 11.

Only recently, DL models have been used in computational biology. For instance, in 34 binding sites of RNA‐binding proteins were predicted using DBNs. For their analysis, they used different types of RNA data to make the predictions. Specifically, they used the primary sequence, the secondary structure, and the tertiary structure of RNAs as input data. Another interesting fact of their analysis is that they used a multimodal DBN, whereas the input comes from multiple separate layers which are correlated with each other, as is the case for the different types of RNA data. Another example study used a deep convolutional network for predicting protein binding on DNA and RNA sequences 20.

Considering the brief history of DL models for general applications and specifically for problems in computational biology, there is currently no verdict about the best DL model for gene expression data. From the available information of previous studies, it looks that RNNs are not the best choice for analyzing gene expression data, because RNNs have been used mainly for sequential data with a correlation structure of nearby input data. Gene expression data do not possess such a sequential ordering and, hence, lack the properties of sequential data entirely. Similar arguments can be raised against CNNs 20. This leaves MLPs and DBNs as potential candidates, because both models have been used successfully in versatile tasks. For our study, we decided to use DBNs and autoencoders as DL models because among the few conducted studies in computational biology some utilize DBNs and our study can further enrich the literature to come to a more complete understanding of DBN for genomics data.

In the following sections, we discuss DBNs in more detail, and then, we study their abilities in isolation and combination with SVMs.

### Deep belief networks

Neural networks have been studied since many years 35, 36, 37, but recently, they gained new interest due to methodological progress in DL 10. For our analysis, we are using DBNs. DBNs use, first, a RBM to initialize the model and then a supervised method for tuning of the parameters 32. These steps are called pretraining and fine‐tuning. For fine‐tuning, the SGD and the basic backpropagation (Bprop) algorithm are commonly used. In addition, we are using the Rprop algorithm, which is a faster variation of the basic backpropagation algorithm.

### Unsupervised pretraining

Neural networks can be trained via purely supervised learning methods; however, a suitable initialization of the model parameters, that is, the weights and biases, can make the learning faster and improve the performance 11. The introduction of the RBM for an unsupervised initialization of the parameters 10, 38 allowed the training of deep architectures that achieved better performance than shallow architectures.

Pretraining of DBNs consists of stacking RBMs, so that the next RBM in a chain is trained using the previous hidden layer as its visible layer, in order to initialize parameters for each layer. It can be shown that this is an efficient approach 39. The choice of how many layers are trained and in what order can be decided freely. For example, the last layer can be trained first and then after a number of epochs the remaining preceding layers 10. The RBM model we study uses binary units and the contrastive divergence (CD) algorithm, a method for approximating log‐likelihood of the RBM.

### Supervised fine‐tuning

After the neural network parameters for each layer – the weights *W* and the biases **b** – have been initialized by RBMs, the parameters can then be tuned more in order to improve the model further. This second stage of DBN learning is called fine‐tuning, which uses the class‐label information of the training data set that was omitted in pretraining.

We want to build models that can fit new samples well, that is, generalize well. This requires mathematical optimization. We achieve this by minimizing an error function (sometimes called loss function). The mean squared error (MSE) is given by:

(1) $$E=\frac{1}{n}\sum_{i=1}^{n}{\|o_{i}-t_{i}\|}^{2}.$$

In Eqn (1), **o**_*i*_ = *f*(**x**_*i*_) is the *i*th output from the network function $f:R^{m}\to R^{n}$ given the *i*th input **x**_*i*_ from the training set $D=D_{\mathrm{train}}=\left\{\left(x_{1},t_{1}\right),\ldots \left(x_{l},t_{l}\right)\right\}$ and **t**_*i*_ is the desired (target) output.

Similarly to maximizing the log‐likelihood of RBM via gradient ascent, we use gradient descent to find the parameter configuration that minimizes the error function.

(2) $$\theta^{\left(t+1\right)}=\theta^{\left(t\right)}-\Delta \theta^{\left(t\right)}=\theta^{\left(t\right)}-\overset{\Delta \theta^{\left(t\right)}}{\overbrace{\eta \frac{\partial }{\partial \theta^{\left(t\right)}}(\sum_{i=1}^{l}E\left(v_{i}|\theta^{\left(t\right)}\right))}}\overset{\text{Regularization}}{\overbrace{-\lambda \theta^{\left(t\right)}+\nu \Delta \theta^{\left(t-1\right)}}}$$

Here, the parameters are the learning rate η, the weight‐cost λ, and momentum ν.

Usually, the gradient is not calculated using the whole training data set D at once, but instead via SGD with smaller mini‐batches. For estimating the gradient of the error function with respect to the weights and biases in each hidden layer and output layer, the backpropagation algorithm is the standard approach for this 11.

Let us denote ${a_{i}}^{l}$ the activation of the *i*th unit in the *l*th layer (*l* ϵ {2, …, *L*}), $b_{i}^{t}$ the corresponding bias, and $w_{\mathrm{ij}}^{l}$ the weight for the edge between the *j*th unit of the (l − 1)th layer and the *i*th unit of the *l*th layer. If the neuron has an activation function, φ, then the activation of the *l*th layer with the (l − 1)th layer as input is $a^{l}=\varphi \left(z^{\left(l\right)}\right)=\varphi \left(w^{\left(l\right)}a^{\left(l-1\right)}+b^{\left(l\right)}\right)$. The following four equations can be derived, see 40:

(3) $$\left\{\begin{array}{c} \delta^{\left(L\right)}=\nabla_{a}E*\varphi^{\prime }\left(z^{\left(L\right)}\right)\\ \delta^{\left(l\right)}=\left({\left(w^{\left(l+1\right)}\right)}^{T}\delta^{\left(l+1\right)}\right)*\varphi^{\prime }\left(z^{\left(l\right)}\right)\\ \frac{\partial E}{\partial b_{i}^{\left(l\right)}}=\delta_{i}^{\left(l\right)}\\ \frac{\partial E}{\partial w_{\mathrm{ij}}^{\left(l\right)}}=x_{j}^{\left(l-1\right)}\delta_{i}^{\left(l\right)} \end{array}\right..$$

In Equation (3), **δ** ^*L*^ is the vector of errors of the output layer (*L*), **δ** ^*l*^ of the *l*th layer, * is the elementwise product of vectors, and φ′ derivative of the activation function. Thus, the activation function is required to be differentiable. The gradient of the error with respect to the activations for the output layer is:

(4) $$\nabla_{a}E=\{\frac{\partial E}{\partial a_{1}^{\left(L\right)}},\ldots ,\frac{\partial E}{\partial a_{k}^{\left(L\right)}}\}.$$

For instance, for the MSE one obtains $\frac{\partial E}{\partial a_{j}^{\left(L\right)}}=\left(a_{j}-t_{j}\right)$.

Using the previous definitions, we can write a pseudocode for the backpropagation algorithm that is presented in Algorithm 1 40. The estimated gradients from Algorithm 1 are then used to update the biases and weights in SGD Eqn. (2). More updates are performed using mini‐batches until the training data have been used entirely.

Algorithm 1 **Backpropagation algorithm (Bprop).**

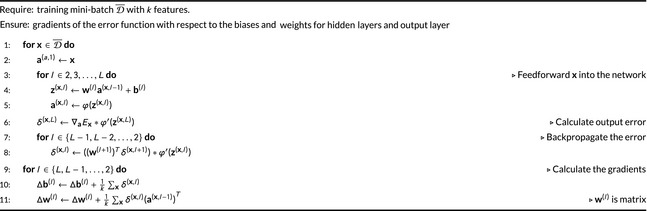

The Rprop algorithm is a modification of the backpropagation algorithm that was originally introduced to speed up the basic backpropagation (Bprop) algorithm 25. Furthermore, there exist at least four different versions of Rprop 41 (Rprop, iRprop^−^, Rprop^+^ and Rprop− (all are supported by the darch package 42)). However, previous studies have shown that the iRprop^+^ algorithm is faster than Bprop and performing best 41.

**Figure 1:**
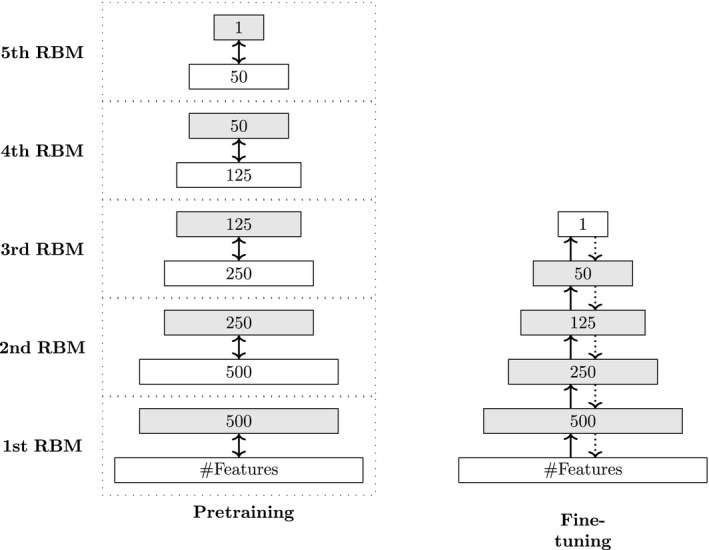
Stages of DBN learning. Two stages of DBN learning. The two edges in fine‐tuning denote the two stages of the backpropagation algorithm: the input feedforwarding and the error backpropagation.

It has been shown that the backpropagation algorithm with SGD can learn good neural network models even without a pretraining stage, when appropriate activation functions are used, and an adequate amount of data are available for the training 11. In Fig. 1, we show an overview of the overall DBN learning procedure.

### Network architecture

At present, there does not seem to be a general consensus among DL researchers about the shape of the architecture of a neural network. In some studies, a decreasing architecture is used 39, whereas others use an increasing architecture 43 or even a constant architecture 44. For this reason, we tested a vast number of different architectures to find the best one for a given constellation. In the results section, we provide more information about the architectures we studied.

For ANNs, the last output layer can be of arbitrary size, but for a binary classification, a good choice is either one node or two nodes. If the activation function is chosen to be the logistic function, the values are in the range [0, 1]. The outputs are then set to be *t*_i_ ϵ {0, 1} or **t**_*i*_ ϵ {*p*, 1 − *p*} with *p* ϵ [0, 1]. If we use the former form, the predicted class is the single output node value rounded, that is, 0 or 1. If we use the latter form, the predicted class is the index of the output vector yielding the higher value. From studies, we observed that the difference between both was negligible.

### Combining deep networks with support vector machines

The idea when combining deep networks with SVMs is to utilize the neural network as a representation learner compressing the original input vector. In this way, the SVM can utilize processed information. In our study, we investigate the influence of different types of deep neural network representations on the combination with a SVM. Specifically, we study RBMs, DBNs, and autoencoders. Fundamentally, all of them perform a dimensionality reduction, since they gradually transform the original representation into higher level representations.

**Figure 2:**
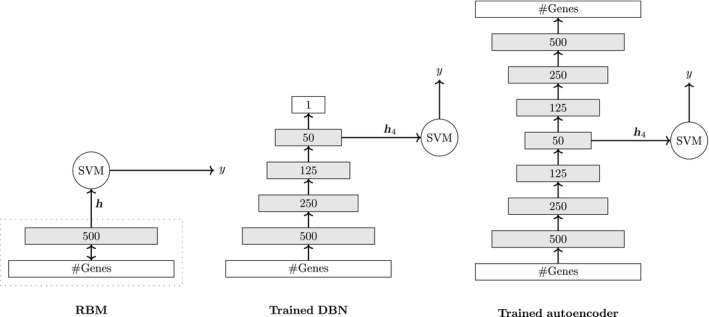
Combining DL representations and SVM. Three ways of combining three types of deep neural network representations with a SVM.

Regarding the choice of the input layer for the SVM, there are different possibility. For instance, for DBNs we can use the last hidden layer with as an input for the SVM. For autoencoders, a good choice is to use the code layer as input. In Fig. 2, we give three examples that show how a deep neural network can be combined with a SVM. As one can see, the combination is simple. In the analysis section, we will present results for many different configurations.

In Algorithm 2, we show pseudocode for the training and testing of the combined classifier. Here, the models $\hat{\mathrm{DBN}}$ and $\hat{\mathrm{SVM}}$ denote the learned classifiers and $\hat{\mathrm{DBN}}$(*i*‐th hidden layer of DBN|*X*_training_) is a mapping from an input vector, given by *X*_training_ to an output, which is defined as the *i*‐th hidden layer of the DBN. These steps summarize the visualization shown in Fig. 2.

Algorithm 2 **Combining a DBN with a SVM.**

**Table:** 

**Input:** training data *X*_training_, training labels *Y*_training_, test data *X*_test_
**Output:** predicted test labels $Y_{\mathrm{test}}^{\prime }$
1: Train DBN model with *X*_training_ and *Y*_training_ → $\hat{\mathrm{DBN}}$
2: Perform feature extraction with $\hat{\mathrm{DBN}}$ for *X*_training_: map each sample from *X*_training_ to the model and use the values from the *i*‐th hidden layer as output $\to X_{\mathrm{training}}^{i}=\hat{\mathrm{DBN}}$ (*i*‐th hidden layer of DBN |*X*_training_)
3: Train SVM model with $X_{\mathrm{training}}^{i}$ and $Y_{\mathrm{training}}\to \hat{\mathrm{SVM}}$
4: Map each sample from *X*_test_ via $\hat{\mathrm{DBN}}$ to an output $\to X_{\mathrm{test}}^{i}=\hat{\mathrm{DBN}}$ (*i*‐th hidden layer of DBN | *X*_test_)
5: For each sample make a prediction for $X_{\mathrm{test}}^{i}$ via $\hat{\mathrm{SVM}}$ to obtain $Y_{\mathrm{test}}^{\prime }$

### Software and hardware

All calculations were carried out in r. The r package darch (versions 0.9.1 and 0.10.0) 42 provided the DL methods, that is, DBNs and the autoencoders. The r package e1071 (version 1.6–7) 45 provided the SVMs including LIBSVM. For our analysis, we used the Tampere center for scientific computing providing the local grid computing resources (TUTGrid).

## Results

For our analysis, we use two DNA microarray data sets, one from breast cancer and one from IBD. In the following two sections, we provide a brief description of both.

### Breast cancer

The breast cancer DNA microarray data we are using for our analysis are from 46. They generated gene expression of lymph‐node‐negative primary breast cancer patients with Affymetrix Human U133a GeneChip. The data can be accessed from the Gene Expression Omnibus (GEO) database, accession number GSE2034.

The data set consists of 286 samples for which raw CEL files are available. We processed the raw data with the affy r‐package 47 for preprocessing. Robust multi‐array average was used for the background correction, quantile normalization for removing any systematic trends arising from the microarray technology and median polish for summarization of the expression values.

**Figure 3:**
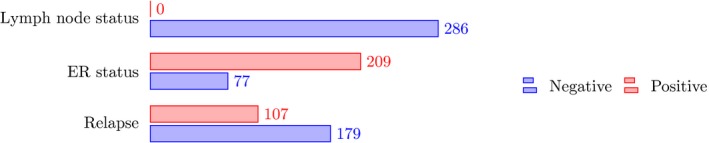
Clinical parameters of the GSE2034 data set. Overview of the clinical parameters of the breast cancer data (GSE2034) 46.

The data set includes the following clinical patient parameters: lymph node status (all negative), relapse (yes or no), estrogen receptor status (ER+ or ER). The clinical parameters are summarized in Fig. 3.

### Inflammatory bowel disease

The second data set we are using provides DNA microarray data for IBDs 48. In total, it consists of 127 samples: 26 UC, 59 Crohn's disease (CD), and 42 normal patients. The data are accessible via GEO, accession number GSE3365. The array used was a Affymetrix Human Genome U133A Array. For these data, no raw CEL files are available in GEO, but the data available are preprocessed with the mas 5.0 algorithm. We transformed the data into a logarithmic scale.

### Performance assessment

For assessing the performance of the classifiers, we use the following error measures.

(5) $$\text{Accuracy (Acc)}=\frac{\text{TP+TN}}{\text{TP+FP+FN+TN}}$$

(6) $$\text{True positive rate (TPR) or sensitivity}=\frac{\text{TP}}{\text{TP+FN}}$$

(7) $$\text{True negative rate (TNR) or specificity}=\frac{\text{TN}}{\text{TN+FP}}$$

(8) $$\text{Error rate}\;\left(E\right)=\frac{\text{FP+FN}}{\text{TP+FP+FN+TN}}.$$

These values can be obtained from the contingency table providing information about TP, TN, FP, and FN 49.

For assessing the variability in the data and for estimating the standard error of the error measures, we are using cross‐validation (CV). CV is the gold standard approach in estimating the prediction error 50. In k‐fold CV, the data set D is once randomly divided into *k* disjoint sets. If |D|=n, then each subset is of size *n*/*k*. The classifier is then trained *k* times, each time using one of the *k* subsets to test the classifier and the remaining *k *−* *1 sets in training. For our analysis, we used *k* = 10, that is, 10‐fold CV.

It is known that the imbalance of classes can lead to problems in the error estimations 51. For this reason, undersampling of the data has been suggested to correct for this imbalance. Some of our data sets are unbalanced. For instance, the breast cancer data set (Fig. 3) has 77 ER+ samples and 209 ER‐ samples. For this reason, we used undersampling to correct for this. Specifically, if the larger class consists of *n*_>_ samples and the smaller class of *n*_<_ sample, we randomly drew *n*_<_ samples from the larger class to balance the classes.

### Regularization

In order to obtain meaningful results for the classification of the disease data, the parameters of our models need to be estimated from training data. In this respect, overfitting is a common problem in supervised learning that can negatively effect the results 52. Due to the importance of this problem, we discuss in this section our counter measures.

**Figure 4:**
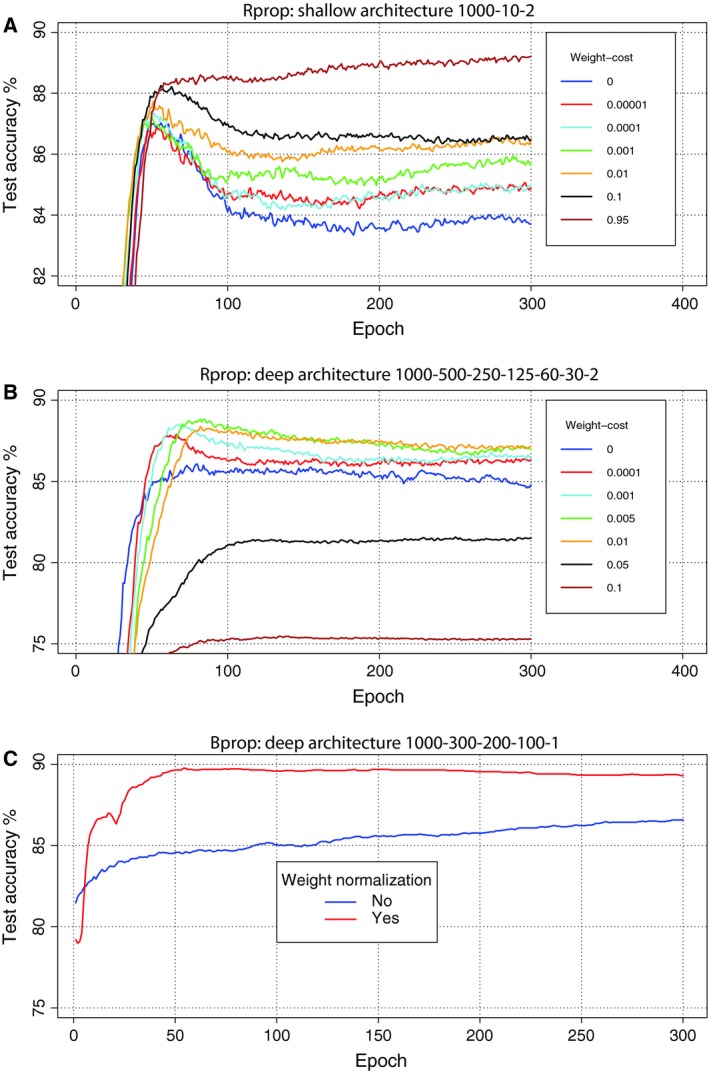
Weight‐decay regularization for Rprop. (A) Shallow architecture. (B) Deep architecture. (C) Weight normalization for Bprop with deep architecture.

In general, regularization is used to adjust parameters for preventing overfitting. The regularization methods we used for the supervised fine‐tuning step are as follows: momentum, weight‐decay, early stopping and weight normalization. For momentum, our analysis found little affect on the performance. Starting with a default value of 0.5 (see Eqn (2)) and switching after 50 epochs to 0.9 worked in general well (results not shown). Weight‐decay is a method for controlling the magnitude of the weights *W* (see Eqn (2)). In Fig. 4A,B, we show two examples for Rprop for breast cancer data (ER status), how tuning the weight‐cost affects the test Acc of a shallow and a deep architecture. We found that especially for architectures with one or two hidden layers, increasing λ values (see Eqn (2)) closer to one help to reduce overfitting by increasing the test set Acc. The comparison indicates that strong weight‐decay regularization is beneficial in reducing overfitting especially in shallow architectures. However, with deep architectures using too high values leads to a negative effect. When the complexity of the network architecture increases, that is, more hidden layers are used, using an overly high value decreased the performance. This may indicate that a strong regularization is needed for the first network interval between the high‐dimensional input and the first hidden layer, but the regularization is not needed as strongly for the subsequent layers. Weight‐decay regularization is also known as L2 regularization 40.

Another frequently used regularization approach in ANN is early stopping 53. For both backpropagation algorithms (Bprop and Rprop), we found that after a certain number of epochs, the test Acc usually started to decrease, although the training Acc increased or stayed at equilibrium. As the examples in Fig. 4 show, early stopping is especially helpful for Rprop, and weight‐decay regularization can reduce the need for using early stopping. We found early stopping to be useful for the breast cancer data. Stopping the training after 90 epoch in general improved the results.

Finally, we tested weight normalization to control the magnitude of the weights. The weights can be normalized so that $\|W_{\cdot j}^{\left(i\right)}{\|}_{2}=1$ holds for each weight matrix *W* ^(*i*)^ and column *j*. Here ${\left\|x\right\|}_{2}=\sqrt{x^{T}x}$ is the L2‐norm of a vector. We found that weight normalization improves the test Acc for Bprop; for an example see Fig. 4C. For Rprop, this normalization increased overfitting, but in combination with early stopping, the performance improved (results not shown).

### Breast cancer

For the breast cancer microarray data set (lymph‐node‐negative patients), we assessed the performance for two different classifications tasks: (a) ER status, comparing ER+ vs ER−, and (b) relapse status, comparing yes vs no.

The results for the best performing DL classifiers, SVMs and other classifiers are summarized in Table 1. For reasons of comparison, we added to Table 1 also results from previous studies 21, 54, 55 (highlighted in blue) that used the same data set. In this table, a* or ^†^ indicates that for this method feature selection was used. For *, 1000 genes having the highest variance were selected and for ^†^, 10 918 genes showing the largest differential expression by utilizing a *t*‐test. For instance, DBN: Bprop* means that a DBN has been trained with the backpropagation (Bprop) algorithm and 1000 genes with the highest variance have been selected as input features for the classifier.

**Table 1:**
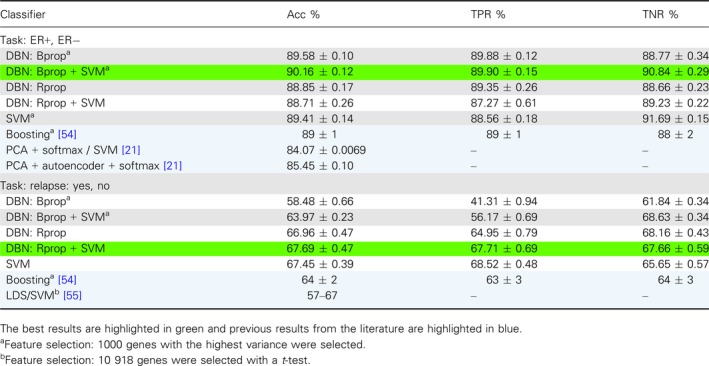
Summary of the results for breast cancer for undersampling the training sets.

Overall, our results show that the prediction of breast cancer relapse is substantially more difficult than predicting the ER status. This is consistent with previous findings 21, 54. For ER status, our DBN with Bprop and SVM obtained the best results when feature selection was used, but other variations with DBN and Rprop and with or without SVM performed good as well. Also, a SVM with feature selection shows good results. All of these results are better than the previously obtained results in 21, 54, see Table 1.

For the relapse task, our DBN with Rprop and SVM without feature selection obtained the best results. For this data set, the differences are in general larger and the standard errors are higher, but also here several other combinations perform similarly well. We want to highlight that a SVM without feature section performs remarkably well. The reference study by 55 used the same SVM library as we, LIBSVM, and their best result is close to ours, 67%. However, the difference is that they selected 10 918 genes with a *t*‐test (significance level of 0.05), hence removing over half of the features. In comparison, our SVM model without feature extraction performs equally. The Boosting result by 54 performs worse compared with our best results and the results by 55.

The results in Table 1 summarize our results from comprehensive investigations of a multitude of different model configurations. Further details of these investigations can be found in Table 2I,II. Table 2I shows results for different network architectures for DBN, and DBN and SVM and different learning algorithms (Bprop and Rprop). The layer highlighted in bold has been used as input for the SVM. In Table 2II, we show results for SVM with radial basis function kernel (RBF) and linear kernel functions. Overall, one sees that there are configurations that do not perform well at all, for example, DBN with Bprop and architecture A‐500‐250‐100‐1 results in Acc = 37.38% for the ER task (see Table 2I). This means that a fine‐tuning effort is needed in order to obtain very good results. The best results in Table 2I,II are italicized. We want to note that all four results are obtained for undersampled data.

**Table 2:**
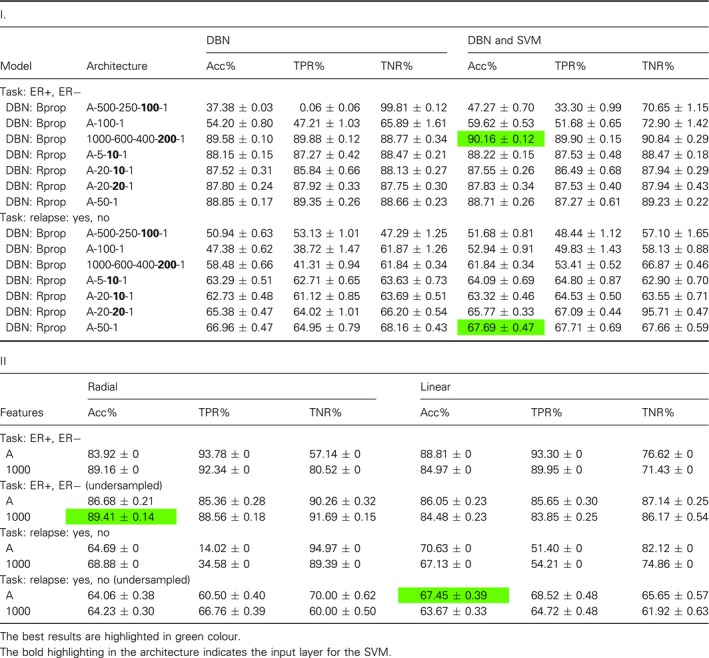
Results for breast cancer data. I: Results for DBN & DBN and SVM. Here A = 22 283 and training sets were undersampled. II: Results for SVM.

There are a few differences between the methods of the reference studies and our methods we would like to mention. Only we and 54 used the undersampling method for the data to correct for imbalanced classes. Another difference is that CV among the studies varied. Two studies used a fivefold CV 54, 55 and one a 10‐fold CV 21.

### Inflammatory bowel disease

The gene expression data for IBD consists of samples for Crohn's disease (CD), UC, and normal samples. We tested all three binary classifications, that is, UC vs CD, UC vs Normal, and CD vs Normal. In addition, we classified them combined, that is, UC vs CD vs Normal. In Table 3, we show a summary of the best results. Similar to our analysis for breast cancer, we performed also here comprehensive investigations for many model parameters and classifier combinations. The results of these analyses are shown in Tables 4I,II and 5, whereas Table 4I shows results for DBN and undersampled data, Table 4II shows results for DBN with Rprop and unbalanced data, and Table 5 shows results for SVM.

**Table 3:**
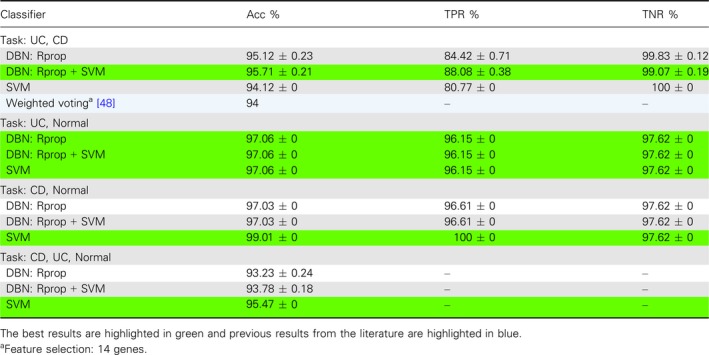
Overall summary of the results for IBD. The results are for unbalanced training sets.

**Table 4:**
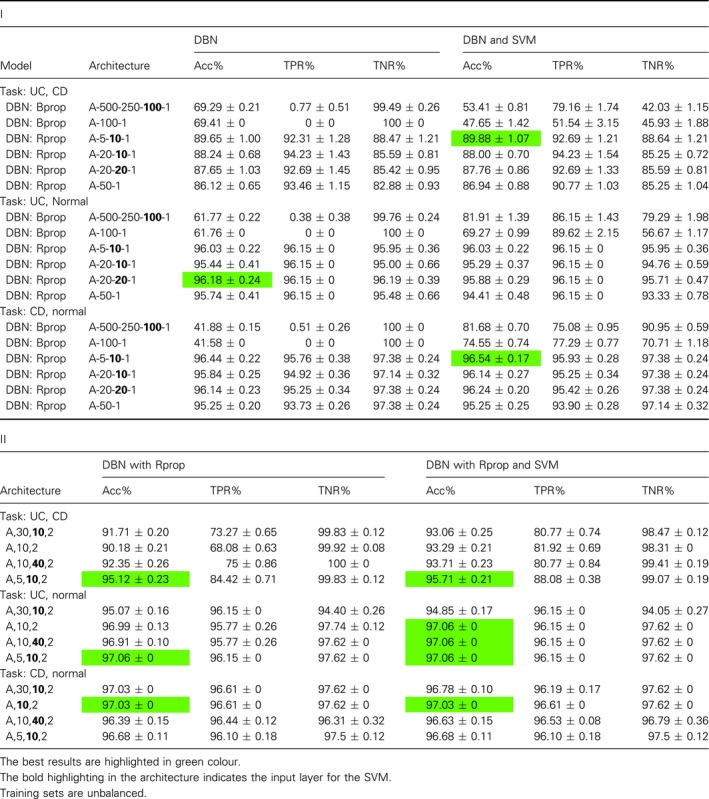
Results for IBD. I. Results for DBN. Here A = 22 283 and undersampled training sets. II: Results for DBN with Rprop and SVM.

**Table 5:**
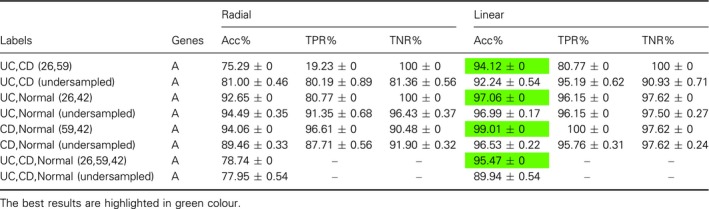
Results for IBD. Results for SVM. Here A = 22 283 and undersampled training sets

Overall, we find that DBN with Rprop and SVM and SVM alone provide the best classification results and the differences are in general smaller than for breast cancer. In 48, a similar analysis was conducted by classifying CD vs UC for the same data set. For this analysis, the weighted voting method from the genecluster 2.0 gene expression analysis software was used. They tested their classifier for different feature sizes of the input vector varying between 1 and 200 genes and found Acc values between 65% and 94%. The highest Acc of 94% was achieved for a feature size of 14 (highlighted in blue in Table 3).

In the original study, the data set was not balanced. For this reason, we performed more tests comparing results for the unbalanced data and balanced data for Bprop and Rprop. The results are shown in the Tables 4I,II. One can see that especially for classifying UC vs CD, there is a large difference showing the influence of unbalanced data. This effect is also observable for the SVM, see Table 5.

It is interesting to note that in general the Bprop algorithm performed poorly compared to Rprop. This seems to be independent of the network architectures and combinations with a SVM.

### Further investigations for the integration of deep learning and SVM

For our next analysis, we focus on the integration of DL and SVM. This results in a new combined classifier that utilizes a hidden layer of the learned neural network as input for the SVM. Hence, the deep neural network is used as a representation learner to serve as a feature selection mechanism for the SVM.

In Table 6I–III, we show results for DBN with Bprop and SVM (Table 6 I), RBM‐learned representations and SVM (Table 6II) and autoencoder‐learned representations and SVM (Table 6III). These results show that Rprop benefited more often from a combination with a SVM than Bprop.

**Table 6:**
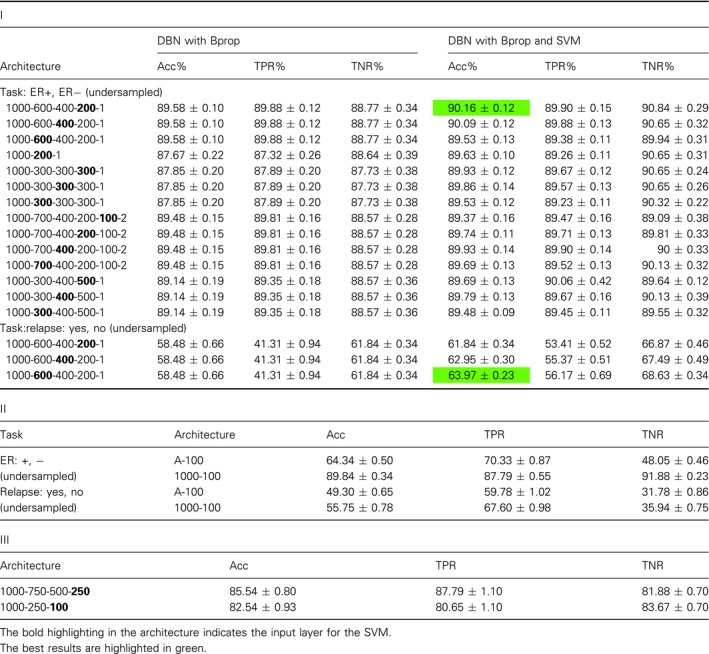
I: Combining DL (DBN with Bprop) and SVM. II: Combining RBM‐learned representations and SVM. III: Combining autoencoder‐learned representations and SVM (ER status). ‐ All results are for breast cancer.

We performed further tests for Bprop to see whether the architecture of the hidden layers has a significant influence. As the results in Table 6I show, the influence of the architecture is moderate regardless of the configuration and the benefit of combining a DBN with Bprop and a SVM is small.

In Table 6II, we show results for the ER status and the relapse task with and without feature selection for RBM and SVM. In principle, RBM can learn a fair representation usable for the SVM. The results for ER status are compatible with the best results in Table 1, but results for relapse status are clearly worse. Furthermore, the results in Table 6II indicate that RBM performed much worse when no feature selection was used (indicated by ‘A’ in the architecture).

In Table 6III, we show results for the autoencoder and SVM for the ER task. We found that the autoencoders performed overall worst, although reasonable good results can be obtained. This is a bit surprising, since the original autoencoder was shown to give significantly better results for learning 2D‐representations from complex data sets with many classes compared to PCA. An explanation for these negative results could be the data requirements of the autoencoder. Specifically, in 39 it has been found that autoencoders require large data sets to function properly.

Our analysis of the autoencoder shows that much more effort would be needed in order to make it competitive. For instance, the same regularization methods as used for Bprop and Rprop could be applied to reduce overfitting in autoencoders, that is, weight normalization, dropout, and weigh‐decay. Both backpropagation algorithms can be used to adjust the parameters in the steepest descent direction, that is, negative of the gradient (see Eqn (2)). Another alternative is to increase them along the conjugate directions. These methods are called conjugate gradient methods. Particularly with autoencoders, conjugate gradient has been shown to yield a better performance than gradient descent 56.

There are previous studies combining a DBN with a SVM, however, outside genomics. For instance, in 57 it has been shown that a SVM with linear kernel performed better than a SVM with RBF kernel, when used with DBNs. Interestingly, the DBN alone outperformed the combined classifiers, but the combined classifiers were still better than a SVM alone. Another study by 16 reported similar results. Their combined classifier performed slightly better than a DBN alone and better than a SVM. Considering these results, it is not surprising that our combined classifiers did not show vast improvements.

## Discussion

Our analyses demonstrated that DBNs can successfully classify complex disorders as represented by gene expression data. Specifically, our results indicate that the top‐performing classifier can predict the ER status of lymph‐node‐negative primary breast cancer with 90% Acc and its relapse with almost 68% Acc. Furthermore, the two principal types of IBD – Crohn's disease and UC – can be distinguished with 95% Acc from each other, and they both can be distinguished from normal patients with over 97% Acc. Moreover, all three classes can be predicted with at least 95% Acc when including them all in the same task.

Overall, the main findings of our comprehensive analysis are as follows. First, no classification method is for all studied conditions always the best. Instead, the best classifier varies in dependence on the conditions. Second, using a SVM alone is the most efficient approach in the sense that the overall usage and set‐up is simple, the needed computational resources are little and the execution time is faster compared to other approaches. We should emphasize that this efficiency is not present in all SVMs but specific to the LIBSVM implementation 27. Third, the general combination of DL with SVM gives always the (marginally) best results. However, there is a considerable effort needed to obtain these results. This includes the finding of the optimal architecture and the learning of the deep network. In addition, large computational resources in form of a computer cluster are required. Fourth, the LIBSVM is capable of dealing very efficiently with high‐dimensional input vectors, either without feature selection or with a moderate selection.

Our results are in contrast to studies in image classification, where DL methods clearly outperformed other classifiers, including SVMs 13. A reason for this difference might be due to the available sample size of the data. Whereas for image classifications ten thousands or even millions of images are available, for genomics studies only hundreds of samples are available. It is important to note that for general genomics studies such a sample size can be considered as high and there is no increase in the near future possible that would increase these sample sizes by four orders of magnitude (a factor of 10 000) that would lead to comparable sample sizes as for image data sets. Hence, genomics data sets will always be much smaller in this sense.

The DL model we analyzed in this study was a DBN. We used a SGD for the optimization of the model in the fine‐tuning stage, and we used two different backpropagation algorithms for minimizing the error, Bprop and Rprop. We found that only Rprop was able to classify data without feature selection, while Bprop needed feature selection. Notably, Rprop worked well even with very small hidden layer sizes. The operability of Bprop seems to be strongly dependent on the RBM‐based pretraining. From performing additional analyses, we found that the reason why Bprop has problems without feature selection is because the pretraining is suboptimal. On the other hand, Rprop appears to be much less dependent on the pretraining, and therefore, it manages to classify the data even without feature selection.

Our second objective was to study how DL representations and SVMs can be combined together. Although some of our results support the conclusion that this combination is beneficial, some of the results show SVMs perform better. The results appear to be task‐specific. Similar results have been obtained previously when combining DBNs and SVMs 16, 57. Neither RBM‐learned representations nor autoencoder‐learned representations seem to be better than DBN‐learned representations, but still provide fair results. The overfitting problem we identified for the autoencoder could be an indicator that the data sets are too small for overfitting methods to work properly. The models consisting of RBM‐learned representations and SVMs support the conclusion that the pretraining has problems without feature selection, and this in turn causes problems for Bprop.

Interestingly, we found that Rprop produces good results with a very small number of hidden layers. Usually, in all DL studies the total number of hidden units is close to the number of input units 10, 14, 17, 19, 43, 58. However, our results show that Bprop in general benefits only little from a larger number of hidden units. A possible reason for this is that the network begins to co‐adapt more; hence, the impact of overfitting increases. In fact, when using Rprop with larger networks we found no beneficial improvement in the performance. This could be also due to data‐specific characteristics, because no previous study investigated gene expression data from genomics.

Finally, we want to remark that DBNs perform an internal feature selection, which enables this method to cope with very high‐dimensional input data. For our analysis, we used varying input sizes between 1000 genes and over 22 000 genes. In order to present a fair comparison between a DBN and other classifiers, it is important to select a classification method that can also handle such high‐dimensional input data without an explicit feature selection step because otherwise performance differences might be attributed to this differing analysis step. As discussed above, the LIBSVM provides such a classification method.

The results obtained in this paper are based on the analysis of two independent data sets from two different diseases. The first data set is from breast cancer and the second from IBD. The sample sizes of both data sets (286 for breast cancer and 127 for IBD) can be considered of reasonable size for gene expression data allowing the application of CV. Importantly, none of our results was data set (disease) specific, but both independent data sets lead to the same overall conclusions regarding the applied classification methods. Hence, one data set could be considered a validation case for the other with respect to our technical results.

## Conclusion

In this paper, we studied the classification of high‐dimensional gene expression data from genomics from breast cancer and IBD. This is an important computational diagnostics task for translational research with possible applications in personalized and precision medicine. We provided a comprehensive analysis of the classification performance of DBNs in dependence on its multiple model parameters and in comparison with SVMs. Based on this analysis, we found the combination of DBN and SVM performs tendentially best, but requires a substantial analysis effort and a thorough technical understanding of DL. In contrast, the LIBSVM implementation of a SVM provides compatible results, which are much easier to attain. Classifiers using only a DBN led to a middle performance but require a similar effort as the combination of DBN and SVM.

Whether other DL classifiers perform differently to DBNs or whether sample expansion methods as, for example, suggested by 22 may lead to different results is left for future studies.

## Conflict of interest

The authors declare that the research was conducted in the absence of any commercial or financial relationships that could be construed as a potential conflict of interest.

## Author contributions

FES conceived the study. JS implemented all scripts and analyzed the data. JS, MD, and FES wrote the paper. All authors approved the final version.
